# Emergency hospital admissions associated with a non-randomised housing intervention meeting national housing quality standards: a longitudinal data linkage study

**DOI:** 10.1136/jech-2017-210370

**Published:** 2018-06-20

**Authors:** Sarah E Rodgers, Rowena Bailey, Rhodri Johnson, Damon Berridge, Wouter Poortinga, Simon Lannon, Robert Smith, Ronan A Lyons

**Affiliations:** 1 Department of Public Health and Policy, University of Liverpool, Liverpool, UK; 2 Health Data Research-UK, Swansea University, Swansea, UK; 3 Data Science Campus, Office for National Statistics, UK; 4 Welsh School of Architecture, Cardiff University, Cardiff, Wales, UK; 5 School of Geography and Planning, Cardiff University, Cardiff, Wales, UK

**Keywords:** housing, longitudinal studies, morbidity, public health, health services

## Abstract

**Background:**

We investigated tenant healthcare utilisation associated with upgrading 8558 council houses to a national quality standard. Homes received multiple internal and external improvements and were analysed using repeated measures of healthcare utilisation.

**Methods:**

The primary outcome was emergency hospital admissions for cardiorespiratory conditions and injuries for residents aged 60 years and over. Secondary outcomes included each of the separate conditions, for tenants aged 60 and over, and for all ages. Council home address and intervention records for eight housing cointerventions were anonymously linked to demographic data, hospital admissions and deaths for individuals in a dynamic cohort. Counts of health events were analysed using multilevel regression models to investigate associations between receipt of each housing improvement, adjusting for potential confounding factors and regional trends.

**Results:**

Residents aged 60 years and over living in homes when improvements were made were associated with up to 39% fewer admissions compared with those living in homes that were not upgraded (incidence rate ratio=0.61, 95% CI 0.53 to 0.72). Reduced admissions were associated with electrical systems, windows and doors, wall insulation, and garden paths. There were small non-significant reductions for the primary outcome associated with upgrading heating, adequate loft insulation, new kitchens and new bathrooms.

**Conclusion:**

Results suggest that hospital admissions can be avoided through improving whole home quality standards. This is the first large-scale longitudinal evaluation of a whole home intervention that has evaluated multiple improvement elements using individual-level objective routine health data.

## Introduction

This paper examines changes in healthcare utilisation following improvements to bring council homes up to a national quality standard.^1 2^ People living in social housing generally have poorer health and other outcomes than the general population.^3^ Poor housing quality has been associated with negative health impacts globally.^4^ It is recommended that policy to reduce health inequalities focuses on the wider determinants of health, including housing.^5 6^ Quantifiable evidence of the health impact and associated costs of healthcare utilisation as a result of poor housing quality is needed to ensure sufficient investment.

A systematic review of improvements to housing found evidence of health benefits following changes to thermal conditions, particularly where these interventions were targeted towards those with chronic respiratory conditions.^7 8^ Evidence of health improvements following interventions that were not specifically targeted at vulnerable groups were less clear; the impacts for everyone in a housing improvement area may conceal health improvements for vulnerable population subgroups. The studies included were predominantly cross-sectional, had relatively limited follow-up periods and used self-reported health in most cases.^9 10^ The review concluded that precise housing conditions and mechanisms causing poor health need further investigation using robust study designs.

Evidence on whole home, housing-led interventions remains unclear.^8^ Multiple elements of a national housing intervention and their impact on self-reported physical and mental health have been evaluated previously using a quasi-experimental design using three waves of cross-sectional survey.^10^ The study reported positive associations with mental health (kitchens and bathrooms, front doors) and physical health (building fabric works) but a negative association on physical health following installation of central heating.^10^ The ability to assess changes in well-being directly from participants rather than waiting for changes in healthcare utilisation has certain advantages but introduces bias and restricts follow-up duration.^8 10^ Previously reported randomised controlled trials (RCTs) have evidenced health benefits using self-reported health data, or reduced healthcare utilisation, associated with an insulation or fall prevention intervention, respectively.^11–13^

We used more than a decade of linked individual-level data to investigate whether emergency hospital admission rates were associated with tenants whose homes were improved to meet national quality standards. To our knowledge there has been no evaluation of multifaceted housing interventions using data linkage and routinely collected data. We have followed the RECORD (REporting of studies Conducted using Observational Routinely collected health Data) statement for reporting.^14^

## Methods

### Study design

A longitudinal panel design was used to study multiple non-randomised housing interventions and associations with healthcare utilisation. Each cointervention was observed at monthly intervals for all tenants for up to 123 months of follow-up. The study design provided, for each cointervention, a counterfactual condition of living in a home that did not meet the housing quality standard (reference group). We compared changes in the health of tenants living in homes that received a housing cointervention during their tenancy (exposure 1) with the reference. We also compared with the reference group changes in the health of tenants living in homes that already met the housing standard (exposure 2). Eight cointerventions were analysed using the monthly healthcare utilisation of all tenants, adjusting for trends in the wider population.

Intervention delivery was determined by the Council according to logistical constraints and was irrespective of health need. In total, there were 2047 possible intervention combinations, and homes were equally likely to receive each of the cointerventions during the decade intervention period.

### Interventions

The type and date of improvements for each housing intervention were sent from the Council to our trusted third party who anonymised these data into the Secure Anonymised Information Linkage Databank.^15^ The eight cointerventions were new (1) windows and doors, (2) kitchens, (3) bathrooms, (4) heating systems, (5) wall insulation, (6) loft insulation, (7) electrical systems and (8) garden paths. The electrical systems cointervention comprised smoke detectors, carbon monoxide detectors, security lights, kitchen and bathroom extractor fans, and internal rewiring.^1 16^

### Participants

Anonymisation of home addresses and intervention data was completed by a trusted third party.^17^ Subsequently, researchers had access to all the anonymised datasets to complete data linkage. Tenants were linked to council homes using the Welsh Demographic Service (WDS) dataset, containing patient-provided address and start and end dates, to determine who lived in each home throughout the study.^18^ Individuals who moved between homes were treated as separate observations; conditions of their previous homes were not taken into account in analysing observations recorded at subsequent addresses. Start and end dates of tenancies were obtained for all residents to censor for migration and death, allowing derivation of a single exposure per person for each cointervention. The primary inclusion criterion was that tenants were registered in one of the homes for at least 60 days between January 2005 and March 2015. The WDS dataset was also used to determine who lived in all other properties in the region using the same rules to create a regional comparator group.

### Variables

Emergency admissions were extracted from the Patient Episode Dataset for Wales, containing complete hospital admissions for all residents of Wales.^19^ Monthly counts of emergency admissions for cardiovascular and respiratory conditions, and injuries for falls and burns, were generated and combined to form the primary outcome (see online supplementary appendix). The secondary outcomes were each of the emergency admissions separated into (1) cardiovascular, (2) respiratory conditions and (3) injuries (falls and burns). The primary outcome of combined admissions was analysed for a subpopulation group of tenants aged 60 years and over, before analysing the secondary outcomes of each admission type separately. The secondary outcomes were then analysed for tenants of all ages.

10.1136/jech-2017-210370.supp1 Supplementary file 1

Potentially confounding variables included age (<25 years old, 25–39, 40–49, 50–59, 60–69, 70–79 and 80+), sex (male, female), comorbidity (0, 1+; see online supplementary appendix, table 1), income deprivation (Welsh Index of Multiple Deprivation Income Domain tertiles, 1=least deprived to 3=most deprived), rurality (Office for National Statistics classification, 1=village and hamlet, 2=town and fringe, 3=urban) and year of study (2005, 2006,…, 2015). Age, comorbidity, rurality and deprivation were updated monthly. Monthly counts of emergency admissions were also derived for the regional comparator group to adjust for background admission trends. Codes for selection of conditions contributing to comorbidities are listed in the online supplementary appendix.

We followed the three groups, comprising one reference and two exposure groups, for each of the eight cointerventions before housing improvements were made from 2005, and during the intervention period from 2007 to 2015. Individuals were categorised differently, and analysed separately, for each of the eight cointerventions.

### Statistical analysis

Separate effect estimates were obtained for each cointervention incorporating counts of emergency admissions within negative binomial models, using random effects (to adjust for autocorrelation of observations for the same individuals over time) and adjusting for potential confounders. Repeated measures multilevel models with 123 monthly observations over time (level 1) nested within tenants (level 2) allowed us to take account of clustering of observations over time. This also helped to handle unbalanced data, where the number of observations varied for individuals, an artefact of dynamic cohorts. In order to adjust for the non-constant observation periods among individuals, we included a log offset of the number of person days observed in each month as an offset in the modelling framework. The observation periods were used to convert results to a person-time rate.^20^ Model coefficients were converted to incident rate ratios (IRRs) to aid interpretation. The IRRs represent the effect estimate of change in outcome intervention groups as compared with the reference group, for each of the different cointerventions. We have discussed these effect estimates in terms of associations in our Results and Discussion sections. All other variables in the model were held constant. We report the 95% CI and exact p values to four decimal places. The results are displayed graphically with their 95% CIs to help with interpretation.

## Results

Over the study period, 32 009 tenant participants were registered to a study home. The study population remained stable over the study period, providing 183 553 person years of follow-up, with an average of 18 031 observed per year. Over 45% of tenants were registered to a study home for the entire observation window, contributing to all 123 monthly records in the study period. Healthcare utilisation was intentionally captured only for the time of their tenancy; therefore, there was no loss to follow-up of outcomes for registered data linked tenants. The regional comparator provided a large number of person years of follow-up to adjust for background trends (231 200 people and 1 628 554 person years). Descriptive statistics are presented in table 1.

**Table 1:** Number and percentage of residents by sociodemographic characteristics for the intervention home tenants and the regional comparator group

	Intervention home tenants		Regional comparator	
	n	%	n	%
	32 009	100	231 200	100
Age group (years)				
<25	13 943	43.6	81 899	35.4
25–39	5435	17.0	43 885	19.0
40–49	2922	9.1	29 393	12.7
50–59	2655	8.3	28 681	12.4
60–69	2774	8.7	22 767	9.8
70–79	2362	7.4	14 895	6.4
80+	1918	6.0	9680	4.2
Sex				
Male	15 173	47.4	114 196	49.4
Female	16 836	52.6	117 004	50.6
Income deprivation quintile				
Most deprived	10 165	31.8	23 137	10.0
More	10 647	33.3	54 856	23.7
Mid	7538	23.5	65 050	28.1
Less	3273	10.2	63 853	27.6
Least deprived	386	1.2	24 304	10.5
Rurality classification				
Urban	17 973	56.1	99 952	43.2
Town	5276	16.5	32 690	14.1
Village and hamlet	8760	27.4	98 558	42.6
Comorbidity status				
No comorbidities	29 426	91.9	219 485	94.9
At least 1 comorbidity	2583	8.1	11 715	5.1

The number of people in the reference and two exposure groups varied for each cointervention. For the electrical systems cointervention, there was one home with missing information about electrical systems (6 tenants, 0.02%), leaving 32 003 tenants out of a total 32 009 study tenants. Tenants who left the home prior to work completion, and a small number living in homes that had not yet received the cointervention by the end of the study period, were assigned to the reference group (n=12 726, 40%). In total, 13 358 tenants (42%) had their electrical systems upgraded during their tenancy and were assigned to exposure 1, and 5919 tenants (18%) whose homes already met the standard before their tenancy began were assigned to exposure 2.

### Outcome data

There were 7296 primary outcome admissions for 27% of the study group tenants aged 60 years and over (table 2). Ten per cent of the study group participants of all ages contributed to 10 524 emergency admissions to hospital for the primary outcome admissions. The adjusted results for the primary outcome population of tenants aged 60 and over are presented in tables 3 and 4, and for tenants of all ages in tables 5 and 6. The adjusted and unadjusted results for tenants aged 60 years and over and tenants of all ages are presented in online supplementary appendix tables 2 and 3.

**Table 2:** Number of admissions and percentage of participants in intervention homes with at least one emergency admission for the primary outcome population (aged 60 years and over) and emergency admissions for each of the separate conditions, and for all ages

Outcome	Aged 60 years and over		All ages	
	n	%	n	%
≥1 primary outcome	7296	27.0	10 524	10.4
≥1 cardiovascular condition	3720	16.9	4661	5.1
≥1 respiratory condition	2849	10.9	4907	5.2
≥1 injury (fall or burn)	700	4.4	956	1.4

**Table 3:** Incidence rate ratios (IRRs) of emergency admissions for tenants aged 60 years and over (exposure 1) for the primary outcome of combined admissions and then for each separate condition: cardiovascular, respiratory and injuries

Combined conditions	IRR	Lower bound	Upper bound	P values	Cardiovascular	IRR	Lower bound	Upper bound	P values
Windows and doors	0.71	0.63	0.81	0.0000	Windows and doors	0.81	0.69	0.96	0.0164
Wall insulation	0.75	0.67	0.84	0.0000	Wall insulation	0.73	0.63	0.85	0.0000
Loft insulation	0.98	0.86	1.11	0.6945	Loft insulation	0.86	0.73	1.02	0.0835
Heating systems	0.91	0.82	1.01	0.0719	Heating systems	0.94	0.82	1.08	0.3886
Kitchens	0.98	0.83	1.17	0.8426	Kitchens	0.91	0.73	1.13	0.3950
Bathrooms	0.93	0.81	1.06	0.2871	Bathrooms	0.94	0.78	1.13	0.5316
Electrical systems	0.61	0.53	0.72	0.0000	Electrical systems	0.80	0.66	0.99	0.0364
Garden paths	0.73	0.64	0.83	0.0000	Garden paths	0.84	0.70	1.00	0.0471

Respiratory	IRR	Lower bound	Upper bound	P values	Injuries	IRR	Lower bound	Upper bound	P values
Windows and doors	0.61	0.49	0.76	0.0000	Windows and doors	0.56	0.40	0.77	0.0004
Wall insulation	0.76	0.62	0.92	0.0055	Wall insulation	0.76	0.57	1.02	0.0700
Loft insulation	1.18	0.95	1.48	0.1376	Loft insulation	1.02	0.73	1.43	0.8867
Heating systems	0.85	0.71	1.03	0.0926	Heating systems	1.01	0.77	1.32	0.9663
Kitchens	1.17	0.86	1.59	0.3257	Kitchens	0.75	0.48	1.17	0.2091
Bathrooms	0.89	0.70	1.13	0.3215	Bathrooms	1.18	0.83	1.66	0.3544
Electrical systems	0.43	0.33	0.57	0.0000	Electrical systems	0.56	0.37	0.85	0.0071
Garden paths	0.62	0.49	0.78	0.0000	Garden paths	0.69	0.49	0.97	0.0352

**Table 4:** Incidence rate ratios (IRRs) of emergency admissions for tenants aged 60 years and over (exposure 2) for the primary outcome of combined admissions and then for each separate condition: cardiovascular, respiratory and injuries

Combined conditions	IRR	Lower bound	Upper bound	P values	Cardiovascular	IRR	Lower bound	Upper bound	P values
Windows and doors	0.80	0.71	0.90	0.0002	Windows and doors	0.91	0.78	1.07	0.2618
Wall insulation	0.73	0.65	0.82	0.0000	Wall insulation	0.72	0.62	0.84	0.0000
Loft insulation	0.93	0.84	1.04	0.2063	Loft insulation	0.87	0.76	1.00	0.0564
Heating systems	1.03	0.88	1.20	0.7357	Heating systems	1.12	0.91	1.37	0.2835
Kitchens	1.09	0.90	1.32	0.3627	Kitchens	1.18	0.92	1.51	0.1931
Bathrooms	0.93	0.79	1.09	0.3690	Bathrooms	0.91	0.74	1.12	0.3733
Electrical systems	0.66	0.56	0.78	0.0000	Electrical systems	0.79	0.64	0.98	0.0295
Garden paths	0.83	0.74	0.92	0.0007	Garden paths	0.94	0.81	1.10	0.4373

Respiratory	IRR	Lower bound	Upper bound	P values	Injuries	IRR	Lower bound	Upper bound	P values
Windows and doors	0.69	0.57	0.85	0.0004	Windows and doors	0.64	0.47	0.86	0.0035
Wall insulation	0.71	0.58	0.86	0.0005	Wall insulation	0.86	0.63	1.16	0.3144
Loft insulation	1.08	0.89	1.31	0.4471	Loft insulation	0.91	0.68	1.22	0.5456
Heating systems	0.92	0.70	1.20	0.5341	Heating systems	1.11	0.71	1.75	0.6411
Kitchens	0.97	0.70	1.34	0.8394	Kitchens	1.04	0.61	1.76	0.8890
Bathrooms	1.01	0.77	1.33	0.9570	Bathrooms	0.66	0.42	1.04	0.0742
Electrical systems	0.51	0.39	0.67	0.0000	Electrical systems	0.76	0.50	1.15	0.1964
Garden paths	0.76	0.62	0.91	0.0036	Garden paths	0.62	0.46	0.82	0.0010

**Table 5:** Incidence rate ratios (IRRs) of emergency admissions for tenants of all ages (exposure 1) for the primary outcome of combined admissions and then for each separate condition: cardiovascular, respiratory and injuries

Combined conditions	IRR	Lower bound	Upper bound	P values	Cardiovascular	IRR	Lower bound	Upper bound	P values
Windows and doors	0.78	0.70	0.87	0.0000	Windows and doors	0.82	0.70	0.96	0.0149
Wall insulation	0.80	0.73	0.87	0.0000	Wall insulation	0.74	0.65	0.85	0.0000
Loft insulation	1.02	0.93	1.13	0.6180	Loft insulation	0.93	0.80	1.08	0.3273
Heating systems	0.92	0.85	1.01	0.0831	Heating systems	0.93	0.82	1.06	0.2864
Kitchens	1.01	0.87	1.18	0.8671	Kitchens	0.95	0.77	1.17	0.6348
Bathrooms	0.99	0.87	1.13	0.8998	Bathrooms	0.99	0.82	1.19	0.9067
Electrical systems	0.66	0.58	0.76	0.0000	Electrical systems	0.79	0.65	0.96	0.0159
Garden paths	0.81	0.73	0.90	0.0001	Garden paths	0.92	0.78	1.09	0.3396

Respiratory	IRR	Lower bound	Upper bound	P values	Injuries	IRR	Lower bound	Upper bound	P values
Windows and doors	0.76	0.65	0.89	0.0007	Windows and doors	0.70	0.52	0.93	0.0152
Wall insulation	0.82	0.72	0.94	0.0042	Wall insulation	0.82	0.63	1.06	0.1293
Loft insulation	1.09	0.95	1.27	0.2246	Loft insulation	1.01	0.76	1.33	0.9694
Heating systems	0.93	0.81	1.07	0.2927	Heating systems	0.94	0.73	1.21	0.6480
Kitchens	1.11	0.87	1.43	0.3933	Kitchens	0.82	0.53	1.27	0.3699
Bathrooms	0.93	0.75	1.15	0.5099	Bathrooms	1.27	0.90	1.81	0.1778
Electrical systems	0.60	0.48	0.74	0.0000	Electrical systems	0.54	0.36	0.81	0.0030
Garden paths	0.74	0.63	0.87	0.0002	Garden paths	0.81	0.60	1.10	0.1786

**Table 6:** Incidence rate ratios (IRRs) of emergency admissions for tenants of all ages (exposure 2) for the primary outcome of combined admissions and then for each separate condition: cardiovascular, respiratory and injuries

Combined conditions	IRR	Lower bound	Upper bound	P values	Cardiovascular	IRR	Lower bound	Upper bound	P values
Windows and doors	0.91	0.82	1.00	0.0516	Windows and doors	0.95	0.82	1.10	0.4893
Wall insulation	0.90	0.82	0.98	0.0171	Wall insulation	0.80	0.70	0.92	0.0013
Loft insulation	1.01	0.93	1.09	0.8678	Loft insulation	0.91	0.81	1.03	0.1517
Heating systems	1.23	1.10	1.38	0.0003	Heating systems	1.21	1.01	1.44	0.0363
Kitchens	1.02	0.86	1.20	0.8152	Kitchens	1.05	0.83	1.32	0.7053
Bathrooms	0.93	0.80	1.07	0.3128	Bathrooms	0.89	0.72	1.09	0.2613
Electrical systems	0.79	0.69	0.90	0.0005	Electrical systems	0.83	0.68	1.01	0.0564
Garden paths	0.86	0.79	0.94	0.0008	Garden paths	0.99	0.86	1.14	0.8909

Respiratory	IRR	Lower bound	Upper bound	P values	Injuries	IRR	Lower bound	Upper bound	P values
Windows and doors	0.88	0.76	1.02	0.0910	Windows and doors	0.83	0.63	1.09	0.1859
Wall insulation	0.95	0.83	1.09	0.4578	Wall insulation	0.87	0.67	1.13	0.3072
Loft insulation	1.11	0.98	1.25	0.1082	Loft insulation	1.00	0.79	1.28	0.9872
Heating systems	1.31	1.11	1.55	0.0018	Heating systems	0.92	0.64	1.34	0.6785
Kitchens	0.98	0.75	1.27	0.8603	Kitchens	1.05	0.63	1.75	0.8523
Bathrooms	0.95	0.75	1.20	0.6831	Bathrooms	0.80	0.51	1.25	0.3267
Electrical systems	0.79	0.64	0.98	0.0287	Electrical systems	0.69	0.46	1.03	0.0701
Garden paths	0.80	0.71	0.92	0.0011	Garden paths	0.72	0.56	0.92	0.0103

### Tenants aged 60 years and over

#### Primary outcome, exposure 1

**Figure 1:**
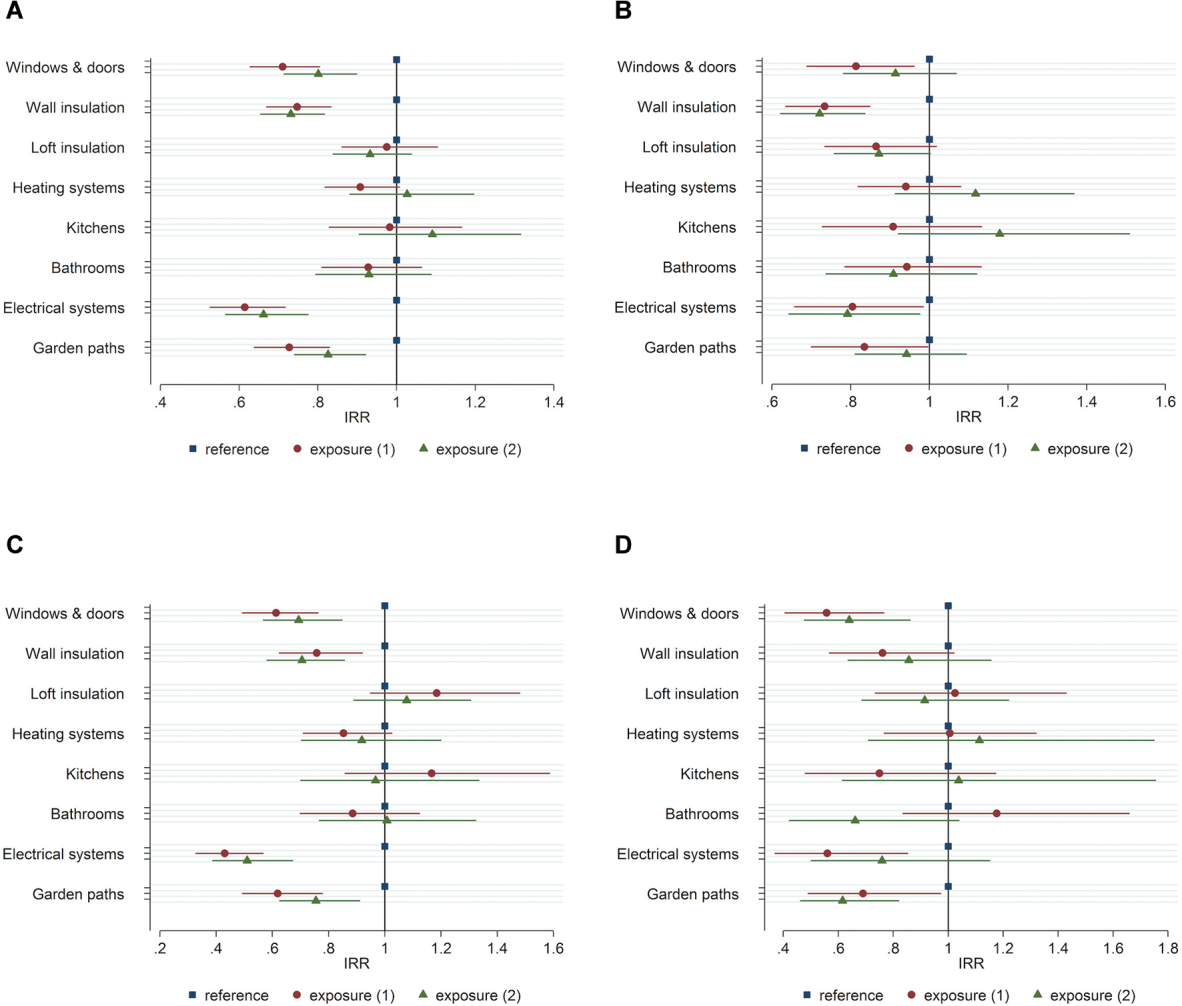
Incidence rate ratios (IRR) of emergency admissions for tenants aged 60 years and over in exposure 1 and exposure 2, compared with the reference group: (A) primary outcome, (B) cardiovascular emergency admissions, (C) respiratory emergency admissions and (D) injury (falls and burns) emergency admissions. Bars represent the extent of a 95% CI.

All cointerventions were associated with a reduction in admissions for tenants aged 60 years and over receiving the upgrades during their tenancy (table 3, figure 1A, circles). The largest association was for the electrical system cointervention (−39%). Large associations were also found for new windows and doors (−29%), wall insulation (−25%) and garden paths (−27%). Smaller associations were found for loft insulation (−2%), heating systems (−9%), kitchens (−2%) and bathrooms (−7%).

#### Primary outcome, exposure 2

Several cointerventions were associated with a reduction in emergency hospital admissions for tenants aged 60 years and over who moved into a home that already met the housing standard for different cointerventions. The largest reduction (−34%) was again associated with electrical systems (table 4, figure 1A, triangles). Large associations were also found for windows and doors (−20%), wall insulation (−27%) and garden paths (−17%). New heating systems and kitchens were associated with small increases in emergency admissions (+3% and +9%, respectively).

#### Secondary outcomes, exposure 1

When we separated our primary outcome into its component conditions and repeated our analyses, we saw widening CIs but a similar pattern overall, with most cointerventions associated with reductions in admissions (table 3, figure 1B–D, circles). The largest association was a 57% reduction in respiratory condition emergency admissions with the electrical system cointervention. Heating systems were associated with a smaller 15% decrease in respiratory admissions. Increased respiratory admissions were associated with loft insulation (+18%) and kitchens (+17%).

#### Secondary outcomes, exposure 2

For cardiovascular admissions there were associated reductions for all cointerventions, apart from those tenants who moved into a home with a heating system or kitchen already meeting the housing standard (+12% and +18%, respectively). Similar to exposure 1 tenants, heating systems were again associated with a small decrease in respiratory admissions (−8%). Injury admissions had associations in different directions for kitchens (−25% for exposure 1, +18% for exposure 2), which reversed for bathrooms (+18% for exposure 1, −44% for exposure 2).

### Tenants of all ages

#### Primary outcome, exposure 1

**Figure 2:**
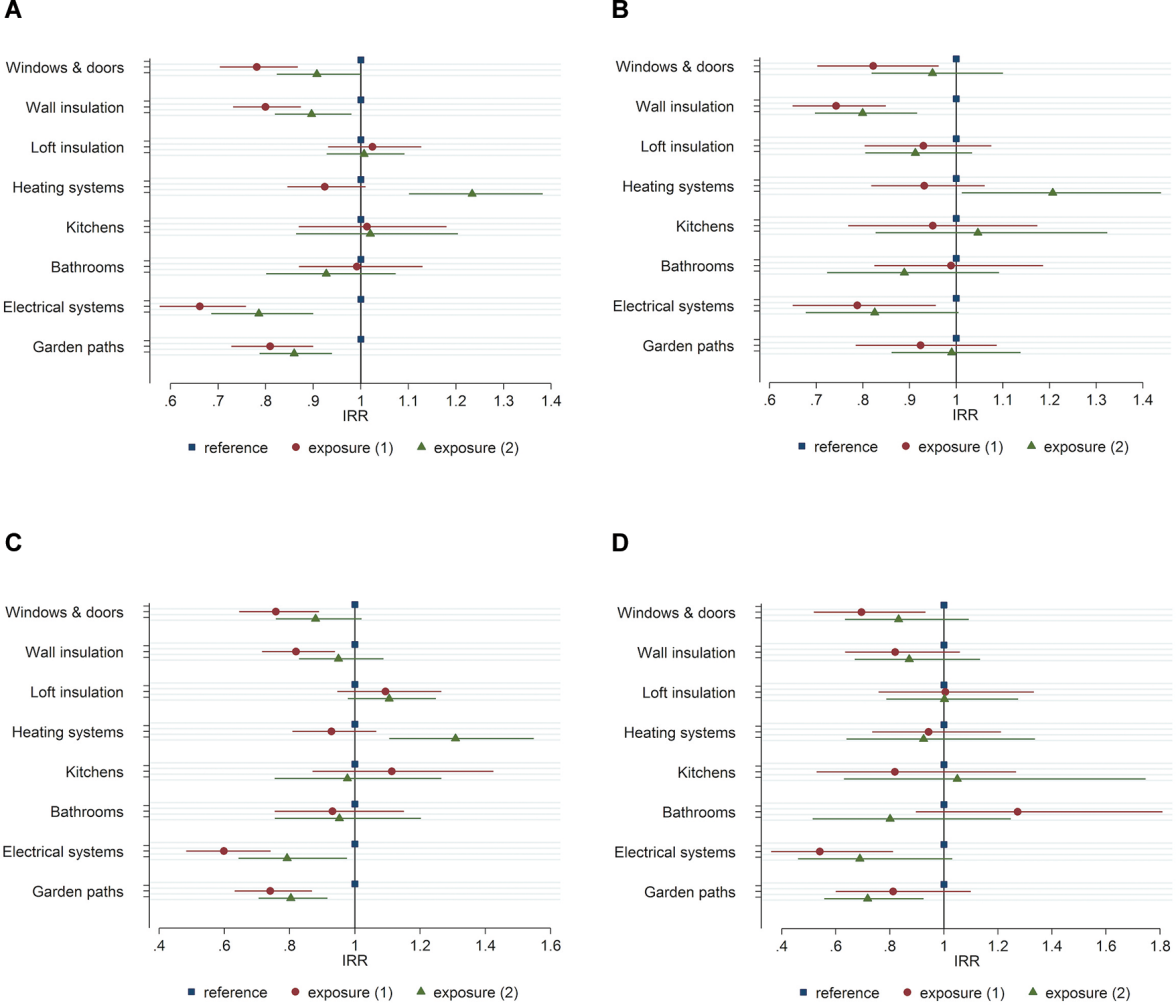
Incidence rate ratios (IRR) of emergency admissions for tenants of all ages in exposure 1 and exposure 2, compared with the reference group: (A) primary outcome, (B) cardiovascular emergency admissions, (C) respiratory emergency admissions and (D) injury (falls and burns) emergency admissions. Bars represent the extent of a 95% CI.

Six out of the eight cointerventions were associated with reduced admissions for current tenants of all ages. Large associations were found for windows and doors (−22%), wall insulation (−20%), electrical systems (−34%) and garden paths (−19%) (table 5, figure 2A, circles). Smaller reductions in hospital admissions were associated with heating systems (−8%) and bathrooms (−1%). In contrast to tenants aged 60 years and over, there were increases in hospital admissions associated with loft insulation (+2%) and new kitchens (+1%) for tenants of all ages.

#### Primary outcome, exposure 2

There were reductions associated with five of the cointerventions for tenants in homes that were already considered up to standard. The reductions were comparable with those for current tenants, with loft insulation associated with a 1% increase and kitchens with a 2% increase (table 6, figure 2A, triangles). The heating system cointervention was notable for associations in different directions; the small (−8%) reduction in admissions for current tenants changed to an increase (+23%) in admissions for tenants who moved into homes with existing heating systems.

#### Secondary outcomes, exposure 1

The degree of association and direction was generally maintained when the cardiovascular, respiratory and injury emergency admissions were separated into their component conditions (table 6). However, exposure 1 tenants living in homes that received new bathrooms were associated with an increase in injury admissions (+27%).

#### Secondary outcomes, exposure 2

There was a large increase (+31%) (figure 2B, triangles) associated with cardiovascular admissions for tenants moving into homes with a heating system already up to standard. Similar associations were found for respiratory admissions (figure 2C). In contrast to a new bathroom, people whose homes had existing bathrooms meeting the housing standards were associated with a reduction in injury admissions (−20%, figure 2D).

## Discussion

The main results of this study were that the housing intervention was associated with a decrease in emergency hospital admissions within the decade-long evaluation period. There was a reduction in the primary outcome of combined emergency admissions for cardiorespiratory conditions and injuries, associated with all cointerventions. When we examined emergency admissions for the separate conditions, the largest reduction was for respiratory conditions. There were also decreases for admissions relating to cardiovascular conditions and fall and burn injuries.

We used the ROBINS-I (Risk Of Bias In Non-randomised Studies - of Interventions) assessment for Non-Randomised Studies of Interventions to assess the study design and highlight strengths and weaknesses.^21^ Lack of participant randomisation means some confounding and selection bias may be present; however, the intervention design was independent from health need and researcher influence. In line with the ROBINS-I assessment tool, we concluded there was moderate bias compared with a well-designed RCT.

In addition to lack of randomisation, other study limitations included a lack of information on the initial housing quality. We were limited to a binary status of meeting or not meeting the housing standard. We recommend that data on initial housing conditions are collected in future, and analyses take this, and the magnitude of improvement variations between homes, into account. Although we treated each cointervention separately in the same statistical model, in practice, there are likely to have been correlations between each, for example kitchen and bathroom improvements. As such the results of associations should be read in terms of their relative magnitude and used to increase understanding of the potential mechanisms for health improvement as a result of a whole home intervention.

The strengths of our study included our use of routinely collected data for a complete housing cohort, removal of recall bias, a long follow-up time and the adjustment for multiple confounders. We had complete data for hospital admissions and death registrations and were able to censor people who moved out of intervention homes, allocating exact exposures to the intervention by the number of days registered to a property. We were able to examine council housing population subgroups using individual-level data, removing the possibility of concealing health improvements within areas for the total council housing population receiving the intervention.^22^ We analysed all people living in council intervention homes for whom we had health records within the databank, which was close to 100%. Our study design allowed us to estimate health utilisation associated with each cointervention, for tenants aged 60 years and over and tenants of all ages, allowing direct comparisons between groups depending on their exposure status.

Our results support evidence from previous RCT studies. A cluster RCT found reduced odds of self-reported health, including wheezing (−43%) and less frequent visits to a general practitioner, and a trend for reduced hospital admissions for respiratory conditions (adjusted OR 0.53, 95% CI 0.22 to 1.29).^23^ A randomised home heating intervention evaluating the health of children with asthma found that having received a more effective non-polluting heater was associated with reduced visits to a general practitioner (adjusted OR 0.40, 95% CI 0.11 to 0.62).^13^ Another study randomised low-income participants to receive a home modification intended to reduce falls. Administrative data were used to evaluate randomly allocated low-income participants to a fall reduction home modification resulting in a 26% reduction in injury rate for the treatment group compared with the control group. When injuries were more narrowly specified to the home modification, the intervention group injury rate was reduced to 39% (adjusted OR 0.61, 95% CI 0.41 to 0.91).^11^

This is the largest, most comprehensive analysis to date of a concentrated programme bringing housing quality to national standards and its associations with healthcare utilisation. This was made possible using data linkage at household and individual levels, and findings highlight a substantial potential for multicomponent housing programmes to improve health overall as evaluated using the proxy of emergency healthcare utilisation.^18 24^ We analysed health service utilisation for population subgroups and several conditions anticipated to change as a result of several housing quality improvements. The results shown here provide evidence of health benefits, indicated by a reduction in emergency admissions to hospital, following improvements in social housing conditions that could be achieved through the implementation of a similar programme of work.

These results have important policy implications. First, they highlight the reduction in health service utilisation through a large decrease in hospital admissions, one of the most expensive components of healthcare costs. Evidencing a reduction in admissions due to housing condition improvements may encourage an integrated housing, health and social care system.^25^ Second, the costs incurred through providing the housing improvements may be partially offset by the reduction in hospital admissions, or would release a number of hospital beds for other admissions. Third, the provision of adequate housing is likely to impact on other health, social and educational outcomes. We recommend that research is undertaken to evaluate if children living in improved homes have improved their school attendance and educational attainment. This would likely lead to improved labour market chances and improved health literacy, and to narrowing inequalities in the long term. Progress is under way to improve social housing to meet the Welsh Housing Quality Standard (WHQS) throughout Wales by 2020, and the results here provide compelling evidence to extend this housing quality standard to all low-income households in maritime temperate regions.

**What is already known on this subject**

- Ecological studies are insufficiently targeted at the residents receiving home improvements to evidence changes in health.
- Two randomised controlled trial (RCT) studies investigated separately home improvements for insulation or fall prevention modifications, and showed improvements in respiratory conditions and fall-related healthcare utilisation, respectively.
- To our knowledge, no studies have evaluated a complex multipart housing intervention using a decade of routinely linked objective data.

**What this study adds**

- Using up to a decade of household improvements linked to individual level data, we found that social housing quality improvements were associated with substantial reductions in emergency hospital admissions for cardiovascular conditions, respiratory conditions, and fall and burn injuries.
- We assessed potential bias in this non-randomised study of a complex intervention and concluded that despite a lack of randomisation, there was only a moderate level of bias compared with a well-designed RCT.
- Our results emphasise the importance of using routine linked data to evaluate interventions as a result of policy or economic changes; large-scale system change data may be used to follow up long-term healthcare utilisation for individuals.
