# Supplementary material for: Emergency hospital admissions associated with a non-randomised housing intervention meeting national housing quality standards: a longitudinal data linkage study
Source: J Epidemiol Community Health. 2018 Jun 20;72(10):896–903. doi: 10.1136/jech-2017-210370 (PMC6161658; doi:10.1136/jech-2017-210370)
Supplement: Supplementary file 1 [file jech-2017-210370supp001.pdf]

Supplemental online appendices

Comorbidity code selection

Comorbidity was calculated using Bottle and Aylin's algorithm that was developed using English National Health Service admission data.[24] We amended the algorithm, opting to not include ICD-10 code C44 (Non-Melanoma Skin Cancer) within the comorbidity algorithm to avoid potential bias due to differential treatment practices, which can be carried out in primary or secondary settings, depending on geographical location in Wales. Additionally, some ICD-10 codes were removed from the SAIL databank for reasons of statistical disclosure of individuals. Therefore, our method was a modified approach. Seventeen categories of comorbidity were used (Appendix, Table 1). Comorbidities were calculated for individuals and updated monthly for entry into the final statistical model.

Appendix, Table 1. ICD-10 codes used in a modified Bottle and Aylin comorbidity score

Group number	Comorbidity	ICD-10 codes	Weights
1	Acute myocardial infarction	I21, I22, I23, I252, I258	5
2	Cerebral vascular accident	G450, G451, G452, G454, G458, G459, G46, I60, I61, I62, I63, I64, I65, I66, I67, I680, I681, I682, I688, I69	11
3	Congestive heart failure	I50	13
4	Connective tissue disorder	M05, M060, M063, M069, M32, M332, M34, M353	4
5	Dementia	F00, F01, F02, F03, F051	14
6	Diabetes without long-term	E101, E105, E106, E108, E109, E111, E115,	3

	complications	E116, E118, E119, E131, E135, E136, E138, E139, E141, E145, E146, E148, E149	
7	Mild or moderate liver disease	K702, K703, K717, K73, K74	8
8	Peptic ulcer	K25, K26, K27, K28	9
9	Peripheral vascular disease	I71, I739, I790, R02, Z958, Z959	6
10	Pulmonary disease	J40, J41, J42, J43, J44, J45, J46, J47, J60, J61, J62, J63, J64, J65, J66, J67	4
11	Cancer	C00-C76, C80, C81-C85, C880, C881, C882, C883, C887, C889, C900, C901, C902,mC91, C92, C93, C940, C941, C942, C943, C944, C945, C947, C95, C96, C97X	8
12	Diabetes with long-term complications	E102, E103, E104, E107,E112, E113, E114, E117, E132, E133, E134, E137, E142, E143, E144, E147	-1
13	Paraplegia	G041, G81, G820, G821, G822	1
14	Renal disease	N01, N03, N052, N053, N054, N055, N056, N072, N073, N074, N18, N19, N25, I12, I13	10
15	Metastatic cancer	C77, C78, C79	14
16	Severe liver disease	K721, K729, K766, K767	18
17	HIV	B20, B21, B22, B23, B24, Z21	2

Bottle, A. & Aylin, P. Comorbidity scores for administrative data benefited from adaptation to local coding and diagnostic practices. J. Clin. Epidemiol. **64,** 1426–1433 (2011).

Hospital admission code selection

Hospital admission outcomes were defined using the Patient Episode Dataset for Wales (PEDW) within the SAIL databank. This dataset contains anonymised hospital admission data for all Welsh based NHS hospitals residents. The primary outcome was emergency admissions to hospital for combined i) cardiovascular and ii) respiratory conditions, and iii) fall and burn injuries. Secondary outcomes were emergency admissions separately for each of the three conditions.

We selected the first episode within a continuous inpatient stay for emergency admissions, which was hypothesised as more likely to indicate the main reason of admission.

The primary diagnosis position was used to determine the reason for admission using ICD-10 classification codes. In cases where the primary diagnosis code recorded was a symptom or other factor influencing health rather than a clinically defined condition we searched subsequent diagnosis code positions within the episode for the first clinically defining diagnosis code to classify admission reason. An exception to this rule was created where no clinically defined conditions were present but the first diagnosis code indicated symptoms involving cardiovascular or respiratory systems.

Cardiovascular conditions were identified where the primary diagnosis code was in the ICD-10 'diseases of the circulatory system' chapter and respiratory conditions were selected where a primary diagnosis was in the ICD-10 'diseases of the respiratory system' chapter.

Falls were identified where the primary diagnosis code was a traumatic injury, combined with a fall code in any subsequent diagnosis position that occurred in the home. Fall codes determined to be reasonably likely to have an association with the home environment were

included, and the fourth character of the fall code was used to select falls occurring in the home.

Records with primary diagnosis burn codes, paired with a cause of burn code in any subsequent diagnosis position that occurred in the home were selected as burn admissions. Cause of burn codes determined to be reasonably likely to have an association with the home environment were included; the fourth character of the cause code was used to select burns occurring in the home.

Emergency admission episodes were aggregated into episode counts for monthly time periods from baseline through follow up.

Appendix Table 2: Unadjusted and adjusted emergency admission outcomes for tenants aged 60 and over

**Emergency admissions for combined conditions for participants aged 60 years and over**	unadjusted						adjusted					
	exposure (1)			exposure (2)			exposure (1)			exposure (2)		
Windows & doors	0.70	(0.61 ,	0.81)	0.80	(0.70 ,	0.92)	0.71	(0.63 ,	0.81)	0.80	(0.71 ,	0.90)
Wall insulation	0.70	(0.61 ,	0.79)	0.73	(0.64 ,	0.83)	0.75	(0.67 ,	0.84)	0.73	(0.65 ,	0.82)
Loft insulation	0.93	(0.80 ,	1.08)	1.00	(0.88 ,	1.13)	0.98	(0.86 ,	1.11)	0.93	(0.84 ,	1.04)
Heating systems	0.82	(0.72 ,	0.93)	0.97	(0.82 ,	1.16)	0.91	(0.82 ,	1.01)	1.03	(0.88 ,	1.20)
Kitchens	1.00	(0.81 ,	1.23)	1.12	(0.90 ,	1.40)	0.98	(0.83 ,	1.17)	1.09	(0.90 ,	1.32)
Bathrooms	0.97	(0.82 ,	1.14)	1.08	(0.89 ,	1.30)	0.93	(0.81 ,	1.06)	0.93	(0.79 ,	1.09)
Electrical systems	0.60	(0.50 ,	0.72)	0.66	(0.54 ,	0.79)	0.61	(0.53 ,	0.72)	0.66	(0.56 ,	0.78)
Garden paths	0.70	(0.60 ,	0.82)	0.82	(0.72 ,	0.93)	0.73	(0.64 ,	0.83)	0.83	(0.74 ,	0.92)

**Emergency admissions for cardiovascular conditions for participants aged 60 years and over**	unadjusted						adjusted					
	exposure (1)			exposure (2)			exposure (1)			exposure (2)		
Windows & doors	0.75	(0.62,	0.89)	0.84	(0.71 ,	1.00)	0.81	(0.69 ,	0.96)	0.91	(0.78 ,	1.07)
Wall insulation	0.67	(0.57,	0.79)	0.67	(0.57 ,	0.79)	0.73	(0.63 ,	0.85)	0.72	(0.62 ,	0.84)
Loft insulation	0.80	(0.67,	0.97)	0.92	(0.79 ,	1.07)	0.86	(0.73 ,	1.02)	0.87	(0.76 ,	1.00)
Heating systems	0.80	(0.69,	0.93)	0.99	(0.80 ,	1.24)	0.94	(0.82 ,	1.08)	1.12	(0.91 ,	1.37)
Kitchens	0.92	(0.72,	1.18)	1.16	(0.89 ,	1.52)	0.91	(0.73 ,	1.13)	1.18	(0.92 ,	1.51)
Bathrooms	0.94	(0.77,	1.16)	1.10	(0.87 ,	1.38)	0.94	(0.78 ,	1.13)	0.91	(0.74 ,	1.12)
Electrical systems	0.77	(0.61,	0.97)	0.75	(0.60 ,	0.94)	0.80	(0.66 ,	0.99)	0.79	(0.64 ,	0.98)
Garden paths	0.74	(0.61,	0.90)	0.87	(0.74 ,	1.02)	0.84	(0.70 ,	1.00)	0.94	(0.81 ,	1.10)

**Emergency admissions for respiratory conditions for participants aged 60 years and over**	unadjusted				adjusted			
	exposure (1)		exposure (2)		exposure (1)		exposure (2)	
Windows & doors	0.66	(0.52 , 0.85)	0.76	(0.60 , 0.95)	0.61	(0.49 , 0.76)	0.69	(0.57 , 0.85)
Wall insulation	0.71	(0.57 , 0.89)	0.76	(0.61 , 0.95)	0.76	(0.62 , 0.92)	0.71	(0.58 , 0.86)
Loft insulation	1.18	(0.92 , 1.51)	1.16	(0.93 , 1.43)	1.18	(0.95 , 1.48)	1.08	(0.89 , 1.31)
Heating systems	0.84	(0.68 , 1.04)	0.99	(0.73 , 1.32)	0.85	(0.71 , 1.03)	0.92	(0.70 , 1.20)
Kitchens	1.21	(0.85 , 1.73)	1.05	(0.73 , 1.51)	1.17	(0.86 , 1.59)	0.97	(0.70 , 1.34)
Bathrooms	0.99	(0.75 , 1.31)	1.14	(0.83 , 1.57)	0.89	(0.70 , 1.13)	1.01	(0.77 , 1.33)
Electrical systems	0.43	(0.31 , 0.58)	0.54	(0.39 , 0.74)	0.43	(0.33 , 0.57)	0.51	(0.39 , 0.67)
Garden paths	0.68	(0.52 , 0.88)	0.81	(0.66 , 1.01)	0.62	(0.49 , 0.78)	0.76	(0.62 , 0.91)

**Emergency admissions for injuries for participants aged 60 years and over**	unadjusted				adjusted			
	exposure (1)		exposure (2)		exposure (1)		exposure (2)	
Windows & doors	0.58	(0.44 , 0.77)	0.66	(0.51 , 0.86)	0.56	(0.40 , 0.77)	0.64	(0.47 , 0.86)
Wall insulation	0.88	(0.67 , 1.14)	0.94	(0.72 , 1.24)	0.76	(0.57 , 1.02)	0.86	(0.63 , 1.16)
Loft insulation	1.06	(0.78 , 1.43)	1.02	(0.79 , 1.32)	1.02	(0.73 , 1.43)	0.91	(0.68 , 1.22)
Heating systems	0.87	(0.68 , 1.12)	0.91	(0.60 , 1.37)	1.01	(0.77 , 1.32)	1.11	(0.71 , 1.75)
Kitchens	0.62	(0.42 , 0.94)	0.88	(0.55 , 1.42)	0.75	(0.48 , 1.17)	1.04	(0.61 , 1.76)
Bathrooms	1.16	(0.85 , 1.60)	0.79	(0.52 , 1.19)	1.18	(0.83 , 1.66)	0.66	(0.42 , 1.04)
Electrical systems	0.61	(0.42 , 0.89)	0.86	(0.59 , 1.25)	0.56	(0.37 , 0.85)	0.76	(0.50 , 1.15)
Garden paths	0.69	(0.51 , 0.95)	0.64	(0.50 , 0.83)	0.69	(0.49 , 0.97)	0.62	(0.46 , 0.82)

Appendix Table 3: Unadjusted and adjusted emergency admission outcomes for tenants of all ages

**Emergency admissions for combined conditions for participants of all ages**	unadjusted						adjusted					
	exposure (1)			exposure (2)			exposure (1)			exposure (2)		
Windows & doors	0.79	(0.70 ,	0.89)	0.87	(0.78 ,	0.97)	0.78	(0.70 ,	0.87)	0.91	(0.82 ,	1.00)
Wall insulation	0.89	(0.80 ,	0.99)	0.94	(0.85 ,	1.04)	0.80	(0.73 ,	0.87)	0.90	(0.82 ,	0.98)
Loft insulation	1.27	(1.13 ,	1.42)	1.30	(1.18 ,	1.42)	1.02	(0.93 ,	1.13)	1.01	(0.93 ,	1.09)
Heating systems	0.65	(0.58 ,	0.72)	0.77	(0.68 ,	0.88)	0.92	(0.85 ,	1.01)	1.23	(1.10 ,	1.38)
Kitchens	0.84	(0.69 ,	1.02)	0.80	(0.65 ,	0.97)	1.01	(0.87 ,	1.18)	1.02	(0.86 ,	1.20)
Bathrooms	1.16	(0.98 ,	1.37)	1.35	(1.13 ,	1.63)	0.99	(0.87 ,	1.13)	0.93	(0.80 ,	1.07)
Electrical systems	0.68	(0.58 ,	0.80)	0.65	(0.55 ,	0.76)	0.66	(0.58 ,	0.76)	0.79	(0.69 ,	0.90)
Garden paths	0.85	(0.75 ,	0.96)	0.98	(0.88 ,	1.08)	0.81	(0.73 ,	0.90)	0.86	(0.79 ,	0.94)

**Emergency admissions for cardiovascular conditions for participants of all ages**	unadjusted						adjusted					
	exposure (1)			exposure (2)			exposure (1)			exposure (2)		
Windows & doors	0.88	(0.74 ,	1.04)	0.90	(0.77 ,	1.05)	0.82	(0.70 ,	0.96)	0.95	(0.82 ,	1.10)
Wall insulation	0.89	(0.77 ,	1.03)	0.86	(0.74 ,	0.99)	0.74	(0.65 ,	0.85)	0.80	(0.70 ,	0.92)
Loft insulation	1.25	(1.07 ,	1.47)	1.32	(1.16 ,	1.50)	0.93	(0.80 ,	1.08)	0.91	(0.81 ,	1.03)
Heating systems	0.56	(0.49 ,	0.65)	0.60	(0.50 ,	0.72)	0.93	(0.82 ,	1.06)	1.21	(1.01 ,	1.44)
Kitchens	0.72	(0.56 ,	0.93)	0.69	(0.53 ,	0.91)	0.95	(0.77 ,	1.17)	1.05	(0.83 ,	1.32)
Bathrooms	1.21	(0.96 ,	1.52)	1.57	(1.22 ,	2.01)	0.99	(0.82 ,	1.19)	0.89	(0.72 ,	1.09)
Electrical systems	0.89	(0.71 ,	1.12)	0.68	(0.55 ,	0.85)	0.79	(0.65 ,	0.96)	0.83	(0.68 ,	1.01)
Garden paths	0.95	(0.79 ,	1.13)	1.09	(0.94 ,	1.26)	0.92	(0.78 ,	1.09)	0.99	(0.86 ,	1.14)

**Emergency admissions for respiratory conditions for participants of all ages**	unadjusted				adjusted			
	exposure (1)		exposure (2)		exposure (1)		exposure (2)	
Windows & doors	0.75	(0.63 , 0.89)	0.86	(0.74 , 1.01)	0.76	(0.65 , 0.89)	0.88	(0.76 , 1.02)
Wall insulation	0.87	(0.75 , 1.01)	1.01	(0.87 , 1.17)	0.82	(0.72 , 0.94)	0.95	(0.83 , 1.09)
Loft insulation	1.30	(1.11 , 1.52)	1.30	(1.14 , 1.48)	1.09	(0.95 , 1.27)	1.11	(0.98 , 1.25)
Heating systems	0.74	(0.63 , 0.86)	1.01	(0.84 , 1.20)	0.93	(0.81 , 1.07)	1.31	(1.11 , 1.55)
Kitchens	1.00	(0.76 , 1.32)	0.87	(0.65 , 1.15)	1.11	(0.87 , 1.43)	0.98	(0.75 , 1.27)
Bathrooms	1.09	(0.85 , 1.38)	1.25	(0.96 , 1.63)	0.93	(0.75 , 1.15)	0.95	(0.75 , 1.20)
Electrical systems	0.56	(0.44 , 0.71)	0.63	(0.50 , 0.79)	0.60	(0.48 , 0.74)	0.79	(0.64 , 0.98)
Garden paths	0.78	(0.65 , 0.93)	0.92	(0.80 , 1.06)	0.74	(0.63 , 0.87)	0.80	(0.71 , 0.92)

**Emergency admissions for injuries for participants of all ages**	unadjusted				adjusted			
	exposure (1)		exposure (2)		exposure (1)		exposure (2)	
Windows & doors	0.70	(0.55 , 0.91)	0.78	(0.62 , 0.99)	0.70	(0.52 , 0.93)	0.83	(0.63 , 1.09)
Wall insulation	1.11	(0.89 , 1.39)	1.12	(0.89 , 1.41)	0.82	(0.63 , 1.06)	0.87	(0.67 , 1.13)
Loft insulation	1.34	(1.05 , 1.72)	1.38	(1.11 , 1.70)	1.01	(0.76 , 1.33)	1.00	(0.79 , 1.28)
Heating systems	0.66	(0.53 , 0.82)	0.56	(0.40 , 0.77)	0.94	(0.73 , 1.21)	0.92	(0.64 , 1.34)
Kitchens	0.54	(0.36 , 0.81)	0.68	(0.43 , 1.10)	0.82	(0.53 , 1.27)	1.05	(0.63 , 1.75)
Bathrooms	1.57	(1.11 , 2.21)	1.15	(0.76 , 1.75)	1.27	(0.90 , 1.81)	0.80	(0.51 , 1.25)
Electrical systems	0.59	(0.41 , 0.85)	0.71	(0.49 , 1.01)	0.54	(0.36 , 0.81)	0.69	(0.46 , 1.03)
Garden paths	0.92	(0.70 , 1.20)	0.82	(0.66 , 1.03)	0.81	(0.60 , 1.10)	0.72	(0.56 , 0.92)
